# Development and evaluation of a family-based graphical tool for longitudinal monitoring of poststroke sequelae

**DOI:** 10.1055/s-0046-1829168

**Published:** 2026-10-23

**Authors:** Marcelo Magaldi Ribeiro de Oliveira, Pollyana Helena Vieira Costa Policarpo, Jose Augusto Malheiros, Arthur Nicolato, Carlos Eduardo Ferrarez, Leonardo Avellar

**Affiliations:** 1 Universidade Federal de Minas Gerais, Faculdade de Medicina, Departamento de Cirurgia, Belo Horizonte MGBrazil.; 2 Universidade Federal de Minas Gerais, Faculdade de Medicina, Departamento de Anatomia e Imagem, Belo Horizonte MGBrazil.

**Keywords:** Stroke, Rehabilitation, Health Communication, Family

## Abstract

**Background:**

Medical information regarding patient health is traditionally managed by healthcare professionals, often leaving patients and families in a passive role during the recovery process.

**Objective:**

To describe and evaluate a family-based functional graphic tool—the Family Observation of Limits and Learning on Well-being (FOLLOW)—designed for the monthly assessment of post-stroke sequelae by nonprofessionals.

**Methods:**

The tool is a color-coded visual graphic (red for no improvement, yellow for stability, and green for improvement) designed for intuitive understanding without specialized training. It evaluates seven domains: motor independence, sleep patterns, cognition and language, family quality of life, patient quality of life, fatigue, and emotional state. This study included 51 patients and assessed the usability and proficiency evaluation among three groups: family caregivers, healthcare professionals, and poststroke individuals.

**Results:**

Significant differences were observed in the proficiency of completing the tool across the groups. Success rates were 94.1% for family caregivers and 100.0% for healthcare professionals, compared with 69.2% for poststroke individuals ( *p* = 0.008). The graphic proved to be easily interpretable for both caregivers and professionals.

**Conclusion:**

The functional graphic is an effective tool when completed by family members or caregivers, though it is less reliable when completed by patients themselves. It provides a practical, longitudinal overview of the patient's home environment and clinical needs, while also serving as a complementary monitoring aid that enables early identification of complications and facilitating prompt intervention.

## INTRODUCTION

Stroke represents one of the most significant causes of long-term disability and psychological distress globally, characteristically disrupting patients' lives without prior warning. 1 2 The sudden onset of neurological deficits often leads to a profound loss of autonomy, severely affecting the individual's social integration and emotional well-being. 3 Because it directly impairs cognitive and motor functions, the overarching impact of a stroke is uniquely devastating among chronic medical conditions, imposing an immediate and complex burden on both the affected individual and modern society. 2 3

Encephalic vascular pathologies, mainly ischemic events, remain a major expense in actual health care, with population aging leading to increasing number of disabled patients. 3 4 Even with acute stroke recent treatments improvements, a considerable number of affect people will be suffering sequels, curbing entire families in some cases. 4 5

The management and reporting of clinical information regarding a patient's health status have traditionally been controlled by healthcare professionals, often relegating patients and their families to a passive role restricted to cross-sectional evaluations during medical appointments. 3 4 This dynamic creates a significant monitoring gap: healthcare professionals interact with chronic patients during less than 1% of their daily routine, leaving family caregivers to manage and observe the remaining 99% of the patient's daily life without standardized tools to systematically record clinical changes. 6 This lack of structured, longitudinal reporting from the home environment prevents timely communication of subtle clinical shifts and delays necessary rehabilitation adjustments.

Although stroke incidence and its associated multidimensional sequelae—encompassing motor, cognitive, and psychosocial domains—are well-documented, 2 4 7 current monitoring paradigms rely heavily on clinical scales designed for periodic hospital or clinic visits. 4 8 These conventional approaches present two major limitations: they fail to capture the continuous, day-to-day functional fluctuations of the patient in their natural home environment, and they rarely integrate multiple disparate domains (such as sleep, fatigue, and family quality of life) into a single, accessible framework. 8 This creates a critical operational gap between hospital discharge and ongoing outpatient rehabilitation.

The Family Observation of Limits and Learning on Well-being (FOLLOW) graphic tool directly addresses this gap by providing a consolidated, low-complexity tracking method that empowers family caregivers to synthesize these distinct functional dimensions into an immediate visual narrative, thereby streamlining home-to-clinic communication.

While several proxy-reported outcome measures (PROMs)—such as the proxy versions of the stroke impact scale (SIS) and caregiver-administered adaptations of the Barthel index (BI)—have been developed to capture the patient's status through a representative's perspective, they often involve extensive questionnaires or strict scoring protocols that can be challenging for continuous, long-term application by laypersons without specific healthcare training. 8 9 Consequently, a distinct gap remains for a simplified, highly intuitive visual tracking tool designed specifically for monthly home assessment. The FOLLOW graphic tool was developed to address this need, utilizing an accessible, color-coded dashboard that allows caregivers to quickly detect shifts across multiple functional domains without the cognitive burden of complex numerical scales.

To address these limitations, this study aims to present the development and preliminary evaluation of a novel family-based functional graphic tool—FOLLOW—designed for the monthly, long-term monitoring of poststroke sequelae by nonprofessionals.

Specifically, this empirical investigation focuses on two main objectives. First, to delineate the multidimensional structure of the FOLLOW graphic tool across its seven core domains (motor independence, sleep patterns, cognition/language, family quality of life, patient quality of life, fatigue, and emotional state). Second, to systematically assess its usability and usability (ease of use and interpretation) by comparing proficiency rates among family caregivers, healthcare professionals, and poststroke individuals.

## METHODS

### Study design and rationale

The present is a methodological and cross-sectional study focused on the development, usability evaluation, and initial usability validation of a novel clinical monitoring tool. Additionally, it incorporates a prospective, longitudinal pilot evaluation tracking patients over a 12-month period to demonstrate the tool's practical utility in the home environment. The study rationale stems from an extensive literature search showing that no single scale concurrently assesses multiple subjective and objective functional domains of poststroke disruptions in a simple, visual, and objective recording that can be managed exclusively by nonprofessionals within the patient's home environment.

### Participants and eligibility criteria

A total of 51 participants were enrolled in this study using a convenience sampling strategy at a university-affiliated neurosurgery and neurological rehabilitation center. The sample was divided into three target groups: healthcare professionals (n = 21), family caregivers (n = 17), and poststroke individuals (n = 13). Participants were recruited face-to-face during routine outpatient follow-up appointments or via direct institutional invitation between February 2023 and August 2023.

### Development of the FOLLOW graphic tool

Two different healthcare professionals, a neurosurgeon, and a physiotherapist, created the graphic. It addresses various aspects as functional motor independence, sleep, cognition and language, emotional state, fatigue and quality of life for both the poststroke individual and their family member. Items considered individual's functionality on their daily activities. It is worth noting that more specific aspects such as muscle strength and balance are implicitly included in the graphic's items and are evaluated through existing scales by health professionals.

### Structure and application protocol

The FOLLOW tool is a visual color-based graphic aiming direct understanding when viewed, without needing specific interpretation. The previously cited six aspects of daily life to be evaluated are expressed according to family and/or patient opinion. Each task register quality of function, with the first letters of the respective activity written in green, yellow, or red, inside a 3-column graphic, meaning good, medium or bad for that function ( Figure 1 ). Every month, a family member or caregiver puts the first letters of evaluated item into the graphic in the correct color, corresponding to how each function is evolving. After some time, the letters can be connected by a line, expressing the graphic registration in a visual manner ( Figure 1 ).

**Figure 1:**
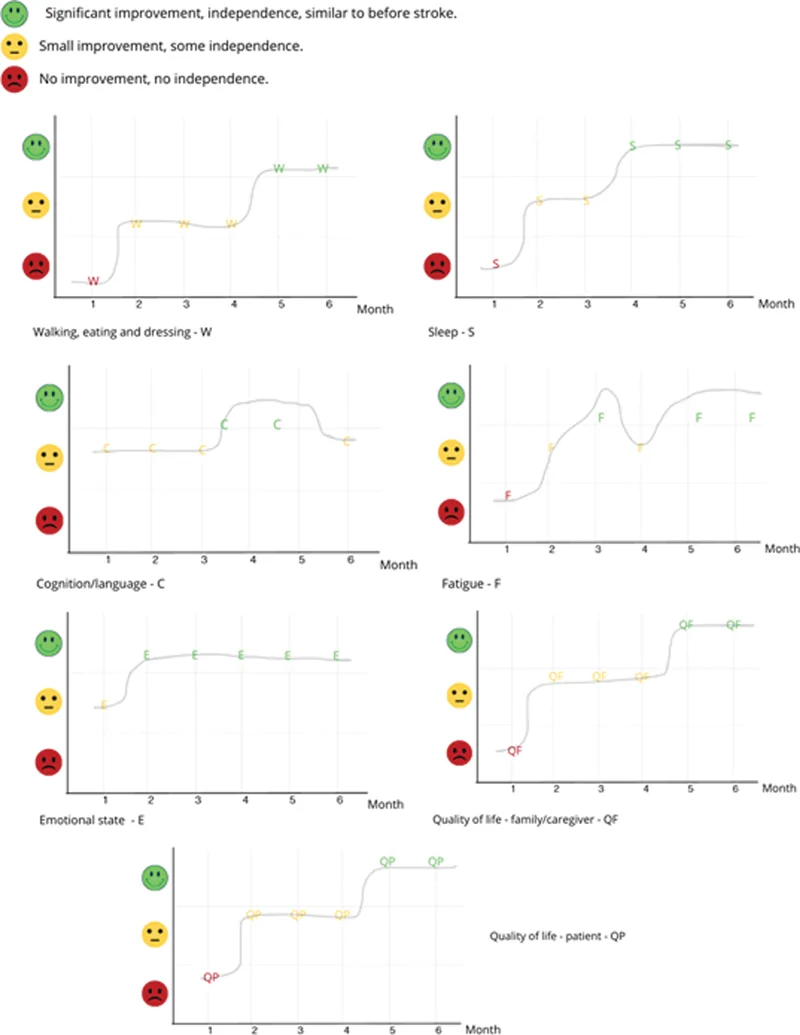
The Family Observation of Limits and Learning on Well-being (FOLLOW) graphic. A color-coded visual tool used for monthly monitoring of poststroke sequelae (Green: improvement; Yellow: stable; Red: no improvement).

### Validation and statistical analysis

To evaluate the usability, face validity, and comprehensibility of the FOLLOW graphical tool, 10 51 individuals as poststroke patients, family/caregivers, and healthcare professionals filled the graphic. Their opinions and responses over a period of 12 months created a picture of evolution of each patient. To evaluate the usability and comprehensibility of the FOLLOW tool, categorical variables (expressed as absolute frequencies and percentages) were analyzed. Proportional differences in tool comprehension and completion proficiency among the three independent target groups (caregivers, healthcare professionals, and patients) were assessed using Pearson's Chi-squared or Fisher's exact tests, as appropriate for small cell frequencies. Statistical significance was set at *p* < 0.05.

Post-hoc power analysis was conducted using G*Power (free) software, version 3.1.9.7. For the comparative analysis of proficiency rates among the three independent groups with a total sample size of 51, assuming a large effect size (Cohen's w = 0.50) and a significance level (Type-I error probability) of α = 0.05, the computed statistical power (1-β) was 0.82, exceeding the standard acceptable threshold of 80%. This confirms that the study sample is sufficiently powered to detect major differences in tool comprehension and usability. All data were analyzed using the IBM SPSS Statistics for Windows (IBM Corp.) software, version 25.0.

## RESULTS

Regarding baseline demographics, the post-stroke patient sample (n = 13) had a mean age of 72 years, with 53% females and a mean time since stroke of 28 months. The family caregivers (n = 17) had a mean age of 46 years (62% female), and healthcare professionals (n = 21) included neurosurgeons, physiotherapists, and nurses, with a mean professional experience of 8 years.

Significant differences were observed regarding the proficiency in completing the FOLLOW tool among the groups. The success rates were 94.1% for family caregivers, 100.0% for healthcare professionals, and 69.2% for poststroke individuals ( *p* = 0.008), as presented in Table 1 . All participants reported that the FOLLOW tool was easy to interpret. Regarding qualitative feedback on missing items, participants noted that the tool could potentially include categories for leisure, entertainment, social relationships, access to caregivers, and healthcare services.

**Table 1:** Usability and comprehensibility of the FOLLOW graphical tool comparing the proficiency of caregivers, healthcare professionals, and poststroke individual regarding tool completion

Questions	Caregiver/Family (n = 17)	Healthcare professional (n = 21)	Poststroke patients (n = 13)	*p* -value
Understood objective	16 (94.1)	21 (100.0)	11 (84.6)	0.110
Understood how to fill out	16 (94.1)	21 (100.0)	9 (69.2)	0.008*
Found it easy	16 (94.1)	21 (100.0)	11 (84.6)	0.110
Useful for you	17 (100.0)	21 (100.0)	13 (100.0)	>0.999
Is there anything missing for you	4 (23.5)	3 (14.3)	0 (0.0)	0.195

Abbreviation: FOLLOW, Family Observation of Limits and Learning on Well-being.

When comparing the usability outcomes between groups, no statistically significant differences were found between family caregivers and healthcare professionals regarding tool comprehension, whereas a significant difference was observed when comparing poststroke patients to the other groups ( Table 1 ). Additionally, the combined group of laypersons (caregivers and patients) showed no statistically significant difference when compared directly to healthcare professionals regarding overall understanding of the objective ( *p* = 0.259), as shown in Table 2 .

**Table 2:** Comprehensibility of the FOLLOW graphical tool comparing laypersons (caregiver/family + patient) and non-laypersons

Questions	Non-layperson (n = 21)	Layperson (n = 30)	*p* -value
Understood objective	21 (100.0)	27 (90.0)	0.259
Understood how to fill out	21 (100.0)	25 (83.3)	0.069
Found it easy	21 (100.0)	27 (90.0)	0.259
Useful for you	21 (100.0)	30 (100.0)	>0.999
Is there anything missing for you	3 (14.3)	4 (57.1)	>0.999

Abbreviation: FOLLOW, Family Observation of Limits and Learning on Well-being.

The longitudinal tracking of 51 poststroke patients over a 12-month follow-up period demonstrated descriptive changes over time in all seven assessed domains, capturing instances of functional improvement, stability, and deterioration across participants.

## DISCUSSION

Due to the absence of simple and comprehensive visual tracking tools targeting a patient's global daily functionality, it became necessary to develop an instrument designed for administration by nonhealthcare professionals. Crucially, the FOLLOW graphical tool is intended to capture data recorded by whoever spends the most time with the patient. Therefore, its primary advantage is the concentration of multiple distinct domains within a single visual framework.

Beyond functioning as a method to monitor functional status over time, it is hypothesized to function as a supplementary visual indicator for families and healthcare providers, potentially highlighting subtle functional fluctuations that might not be captured during standard cross-sectional clinic visits. Whether this structured tracking can actively guide timely clinical decision-making or support the assessment of therapy effectiveness remains a subject for future comparative clinical trials.

All volunteers participating in this research accepted the FOLLOW tool regarding its intended purpose, which is to provide a low-complexity layout to monitor a post-stroke individual's functional status. While various health diaries, digital dashboards, and personalized patient-reported outcome measures exist to track poststroke recovery, many require clinical mediation or focus on isolated functional domains. The FOLLOW tool contributes to this existing literature by consolidating multiple clinical and lifestyle domains into a single, highly structured, color-coded visual layout designed specifically for direct administration by laypersons in the home environment.

Beyond functioning as a method to monitor functional status over time, it serves as a clinical alert for family members and healthcare professionals, highlighting potential fluctuations that might go unnoticed by traditional cross-sectional scales. This structured tracking can also support clinicians in assessing the ongoing effectiveness of rehabilitation therapies. All volunteers participating in this research accepted the FOLLOW tool regarding its intended purpose, which is to provide a low-complexity layout to monitor a poststroke individual's functional status. While various health diaries, digital dashboards, and personalized patient-reported outcome measures exist to track poststroke recovery, many require clinical mediation or focus on isolated functional domains. The FOLLOW tool contributes to this existing literature by consolidating multiple clinical and lifestyle domains into a single, highly structured, color-coded visual layout designed specifically for direct administration by laypersons in the home environment.

When examining measurement properties and intended clinical purposes, a clear distinction emerges between the FOLLOW tool and established neurological scales. For instance, there is no other evaluative clinical scale, to the authors' knowledge, that incorporates sleep patterns, mood, and family quality of life within the same rapid-assessment framework. 8 Although these characteristics are highly subjective and present different psychometric measurement properties than objective motor domains, they often represent the most significant psychosocial barriers to an individual's independent functional recovery after a stroke.

The modified Rankin scale (mRS) remains a widely accepted, ordinal instrument with scores ranging from 0 to 6. 4 9 Its primary measurement property is the categorical, global assessment of hard clinical outcomes and gross functional independence, making it highly effective for survival studies and acute therapeutic trials. However, its categorical structure lacks the granularity required to detect subtle, daily multidimensional changes, omitting specific qualitative aspects of daily functionality.

The BI is closer to the domains addressed by the FOLLOW tool, covering feeding, bathing, dressing, bowel and bladder functions, toileting, transfers, and mobility. 4 8 9 Nevertheless, the BI strictly analyzes activities of daily living (ADL) focused on physical execution. It does not possess measurement properties designed to capture vital qualitative domains such as sleep quality, emotional status, caregiver burden, or cognition/language. Furthermore, both the mRS and BI are validated exclusively as clinician-administered, cross-sectional instruments designed for formal clinical staging during periodic appointments. Neither instrument is optimized for immediate, continuous, and visual longitudinal tracking managed directly by laypersons within the residential environment.

Some participants suggested the integration of specific items to grade leisure, entertainment, social relationships, and access to healthcare services. However, these dimensions are implicitly embedded within the broader domains covered by FOLLOW, except for healthcare services, which fall outside the intended scope of this monitoring tool. Because FOLLOW aims to longitudinally track basic daily functions, users are encouraged to utilize the margins of the graphical sheet to record qualitative nuances or specific contextual notes regarding these omitted items.

Several limitations must be acknowledged in this initial study. First, the sample size during the usability phase was relatively small (n = 51). Second, this study presents a preliminary evaluation of comprehensibility and feasibility, lacking a formal, robust psychometric evaluation—such as structural construct validation or direct criterion comparison against fully validated instruments. Third, several domains embedded within the FOLLOW layout, including sleep patterns, mood, and life satisfaction, are inherently subjective, meaning they rely on qualitative individual perceptions rather than rigid objective metrics.

Finally, because the tool is optimized for administration by nonprofessional family caregivers, it is susceptible to potential caregiver bias; a caregiver experiencing high levels of burnout or emotional fatigue may inadvertently underscore or overscore the patient's actual functional domains. These limitations underscore that, while the FOLLOW tool demonstrated high practical usability, future multicenter studies with larger cohorts are strictly required to establish its psychometric properties, interrater reliability, and control for proxy-reporting biases.

In conclusion the initial evaluation of the FOLLOW graphical tool demonstrates high practical usability, comprehensibility, and feasibility for home-based monitoring, particularly when completed by family caregivers rather than the stroke survivors themselves. The tool offers a structurally low-complexity and visually accessible layout for monthly tracking of multi-dimensional domains. While it does not aim to substitute validated clinical scales, it holds potential as a complementary instrument to provide families and healthcare providers with a continuous qualitative overview of a patient's home environment. Future prospective, controlled clinical trials are strictly required to investigate its definitive clinical applicability and to confirm whether its routine use can actively facilitate timely outpatient interventions.
